# Proteomics Analysis Identified ASNS as a Novel Biomarker for Predicting Recurrence of Skull Base Chordoma

**DOI:** 10.3389/fonc.2021.698497

**Published:** 2021-09-01

**Authors:** Yutao Shen, Mingxuan Li, Yujia Xiong, Songbai Gui, Jiwei Bai, Yazhuo Zhang, Chuzhong Li

**Affiliations:** ^1^Department of Neurosurgery, Beijing Tiantan Hospital, Capital Medical University, Beijing, China; ^2^Beijing Neurosurgical Institute, Capital Medical University, Beijing, China; ^3^Center of Brain Tumor, Beijing Institute for Brain Disorders, Capital Medical University, Beijing, China; ^4^China National Clinical Research Center for Neurological Diseases, Beijing, China

**Keywords:** skull base chordoma, proteomics, asparagine synthetase, recurrence, nomogram

## Abstract

**Background:**

The prognostic factors of skull base chordoma associated with outcomes of patients after surgery remain inadequately identified. This study was designed to identify a novel prognostic factor for patients with skull base chordoma.

**Method:**

Using a proteomic technique, the tumor biomarkers that were upregulated in the rapid-recurrence group of chordoma were screened and then narrowed down by bioinformatic analysis. Finally one potential biomarker was chosen for validation by immunohistochemistry using tissue microarray (TMA). A total of 187 patients included in TMA were randomly divided into two cohorts, the training cohort included 93 patients and the validation cohort included 94 patients. Kaplan-Meier survival analysis was used to assess the patients’ survival. Univariable and multivariable Cox regression analysis were used to identify prognostic factors predicting recurrence-free survival (RFS). CCK-8 assay, clonal formation assay and transwell assay were used to test the effect of asparagine synthetase (ASNS) on the proliferation, migration and invasion in chordoma cell lines.

**Results:**

Among 146 upregulated proteins, ASNS was chosen as a potential prognostic biomarker after bioinformatics analysis. The H-scores of ASNS ranged from 106.27 to 239.58 in TMA. High expression of ASNS was correlated with shorter RFS in both the training cohort (p = 0.0093) and validation cohort (p < 0.001). Knockdown of ASNS by small interfering RNA (siRNA) inhibited the growth, colony formation, migration and invasion of chordoma cells *in vitro*.

**Conclusion:**

This study indicates that high expression of ASNS is correlated with poor prognosis of patients with skull base chordoma. ASNS may be a useful prognostic factor for patients with skull base chordoma.

## Introduction

Chordoma, a rare low-grade malignant tumor that comprises 1-4% of primary bone tumors, arises in the axial bones, and as much as 35% of chordoma locates in the clivus region (1). Chordoma is generally recognized that originates from notochordal remnants (2). The majority of chordoma occurs in adults, aged between 50 and 60, while less than 5% of chordoma occurs in children and infants (3). Although chordoma is a slow-growing and low-grade malignant tumor, effective treatments for curing chordoma are still lacking. In the clivus region, chordoma usually locates close to crucial neurovascular structures, and the tumor can infringe surrounding tissues and vessels, making total resection difficult to accomplish and causing a high recurrence rate. Chordoma is generally considered greatly resistant to conventional chemotherapy and radiotherapy, but recently, photon radiation, proton therapy and targeted therapy have been combined as a comprehensive therapy to prevent chordoma progression (4). However, currently we still have a poor understanding of the molecular biological characteristics and tumorigenesis of chordoma.

Proteomics analysis provides the prospect of searching potential tumor biomarkers and has become a significant technique in the field of cancer research (5, 6). Proteomics technique combined with bioinformatics analysis has a decided advantage in screening and identifying differentially expressed proteins (7), and is successfully used for discovering diagnostic biomarkers (8, 9) and prognostic biomarkers (10, 11), also providing new methods to search therapeutic targets as well as chances to reveal the molecular mechanisms underlying the disease (12, 13).

The asparagine synthetase (ASNS) is an enzyme which catalyzes the conversion of aspartic acid to asparagine (14). This reaction requires glutamate serving as the nitrogen source and proceeds in an ATP-dependent manner (14). The protein of ASNS expresses in different tissues and organs ubiquitously, but except for the exocrine pancreas, the basal expression of ASNS is relatively low in normal tissues (15, 16). Previous studies have reported that ASNS is involved in tumorigenesis in different cancer types. In human melanoma and breast cancer, knockdown of ASNS has been demonstrated to suppress cell growth *via* inducing cell cycle arrest (17, 18). Upregulation of ASNS has been reported to be involved in drug-resistance in prostate cancer and nasopharyngeal carcinoma (19, 20). Moreover, it has been found that the expression of ASNS is associated with the prognosis of patients with hepatocellular carcinoma and ASNS expression is also correlated with the aggressiveness of glioma (21, 22). However, the expression level and underlying involvement of ASNS in skull base chordoma have not been investigated.

In this article, we used a tandem mass tag (TMT) technique combined with bioinformatic analysis to search biomarkers for the prediction of recurrence in skull base chordoma. Proteomic analysis revealed that ASNS was significantly overexpressed in the rapid-recurrence group compared with the slow-recurrence group, and it was confirmed by Western blot assay. We further assessed the expression level of ASNS in skull base chordoma by immunohistochemistry using tissue microarray (TMA) and then investigated its correlation with prognosis especially the time to recurrence after surgery. Our results may provide a novel biomarker for predicting the recurrence of skull base chordoma after surgical resection.

## Materials and Methods

### Patients and Specimens

In this study, frozen tissue samples obtained from 17 patients with skull base chordoma who received surgical resection at Beijing Tiantan Hospital between March 2015 and December 2018 were subjected to proteomics analysis. These patients were followed up by radiographical and clinical examinations in November 2019. Tumor recurrence was confirmed by clinical and imaging findings or histology analysis of specimens from the second surgery. The patients whose recurrence-free survival (RFS) was no more than 6 months and their histopathology images met one of the following criteria: (1) ≥3 mitotic figures in 10 high-power fields; (2) necrosis was present (23, 24), were incorporated into the rapid-recurrence group, and the patients whose RFS was no less than 36 months were incorporated into the slow-recurrence group. All these patients received no radiotherapy after surgery. According to this criterion, 9 patients were enrolled in the rapid-recurrence group and the other 8 patients were enrolled in the slow-recurrence group, the clinicopathological characteristics of these 17 patients were shown in **Supplementary Table 1**. No significant difference was found in age, sex, bone invasion, and extent of resection between the rapid-recurrence group and slow-recurrence group.

Paraffin-embedded tissue samples obtained from 187 patients with primary skull base chordoma who received surgical resection at Beijing Tiantan Hospital between January 2008 and September 2014 were subjected to tissue microarray (TMA). The follow-up information of the 187 patients was updated in November 2019. All patients were randomly divided into two cohorts, the training cohort included 93 patients and the validation cohort included 94 patients. The mean age (± standard deviation) of patients was 40.3 ± 15.9 in the training cohort, and 40.0 ± 14.7 in the validation cohort. The training cohort included 51 males and 42 females, and the validation cohort included 47 males and 47 females. For the extent of resection, the patients with total resection, subtotal resection, and partial resection are 23, 45, 25, respectively in the training cohort; the patients with total resection, subtotal resection, and partial resection are 18, 40, 36, respectively in the validation cohort. This study was approved by the ethics committee of Beijing Tiantan Hospital, Capital Medical University. Informed consent was obtained from all the enrolled subjects, and the study was performed in compliance with the principles governed by the Declaration of Helsinki.

### Mass Spectrometric Detection and Proteomic Analysis

Proteins were extracted from 9 cases of chordoma with short recurrence-free survival and 8 cases of chordoma with long recurrence-free survival frozen tissue samples. Then trypsin digestion was performed, the protein solution was reduced with 5 mM dithiothreitol for 30 min at 56°C and alkylated with 11 mM iodoacetamide for 15 min at room temperature in darkness. The protein sample was then diluted by adding 100 mM TEAB to urea concentration less than 2M. Finally, trypsin was added at 1:50 trypsin-to-protein mass ratio for the first digestion overnight and 1:100 trypsin-to-protein mass ratio for a second 4 h-digestion. After trypsin digestion, peptides were desalted by Strata XC18 SPE column (Phenomenex) and vacuum dried. Peptides were reconstituted in 0.5 M TEAB and processed according to the manufacturer’s protocol for the TMT 10plex kit (Cat No: 90406, ThermoFisher). Briefly, one unit of TMT reagent was thawed and reconstituted in acetonitrile. Equal amount peptides of all samples were taken to make the mix prior to TMT labelling. The peptide mixtures were then incubated for 2 hours at room temperature and pooled, desalted, and dried by vacuum centrifugation. The labelling information is as follows:

**Table:** 

**Group1**	**A1640**	**A1652**	**A1746**	**A1853**	**A1999**	**B1675**	**B1732**	**B1775**	**B1804**	**Mix**
**Label**	126	127N	127C	128N	128C	129N	129C	130N	130C	131
**Group2**	**A2048**	**A2227**	**A2362**	**A1642**	**B1822**	**B1300**	**B1601**	**B802**		**Mix**
**Label**	126	127N	127C	128N	128C	129N	129C	130N		131

A1640, A1652, A1746, A1853, A1999, A2048, A2227, A2362, and A1642 were the Sample ID of samples in the rapid-recurrence group; B1675, B1732, B1775, B1804, B1822, B1300, B1601, and B802 were the Sample ID of samples in the slow-recurrence group.

The peptides were subjected to NSI source followed by tandem mass spectrometry (MS/MS) in Q Exactive™ Plus (Thermo) coupled online to the UPLC. The electrospray voltage applied was 2.0 kV. The m/z scan range was 350 to 1800 for a full scan, and intact peptides were detected in the Orbitrap at a resolution of 70,000. Peptides were then selected for MS/MS using NCE setting as 28 and the fragments were detected in the Orbitrap at a resolution of 17,500. A data-dependent procedure that alternated between one MS scan followed by 20 MS/MS scans with 15.0s dynamic exclusion. Automatic gain control (AGC) was set at 5E4. The fixed first mass was set as 100 m/z. The resulting MS/MS data were processed using the Maxquant search engine (v.1.5.2.8). Tandem mass spectra were searched against the UniProt database concatenated with reverse decoy database. Trypsin/P was specified as a cleavage enzyme allowing up to 2 missed cleavages. The mass tolerance for precursor ions was set as 20 ppm in the First search and 5 ppm in the Main search, and the mass tolerance for fragment ions was set as 0.02 Da. Carbamidomethyl on Cys was specified as fixed modification and oxidation on Met was specified as variable modifications. FDR was adjusted to < 1% and the minimum score for peptides was set > 40. For protein quantification, the ratios of the TMT reporter ion intensities in MS/MS spectra from raw data sets were used to calculate fold changes between samples. Only peptides unique for a given protein were considered for relative quantitation. For each sample, the quantification was normalized using the average ratio of all the unique peptides. Then, protein quantitation was calculated from the median ratio of protein corresponding unique peptides when there were at least two unique peptides in a protein. The average quantitative value of each sample in multiple repetitions was calculated, and then the ratio of the average value between the two cohorts was calculated. This ratio was used as the final differential expression of the comparison group. The differential expression significance *p* value of the protein in the two cohorts was calculated by performing log2 logarithmic conversion of the relative quantitative value of each sample, and then calculating the *p* value using the two-sample two-tailed *t* test method. Two replicates were performed on UPLC-MS/MS for enhancement of protein coverage, and quantified proteins were filtered with manually selected filter exclusion parameters (at least 2 peptides, *p* < 0.05 and expression level differed at least 1.2- or 0.8-fold in the rapid-recurrence group compared to the slow-recurrence group). Bioinformatic analysis was performed focusing on these upregulated proteins in the rapid-recurrence group. The mass spectrometry proteomics data have been deposited to the ProteomeXchange Consortium *via* the PRIDE partner repository with the dataset identifier PXD025894.

### Tissue Microarray

Paraffin-embedded chordoma tissue samples from all 187 patients were assayed by TMA using the Tissue Array MiniCore (ALPHELYS, Plaisir, France). Three pathologists viewed the hematoxylin-eosin stained slides, and the two most representative 2 mm cores from every tissue slide were selected and removed to a new slide to build the TMA. The 4 mm sections from the TMA were cut using Leica RM 2135 Rotary Microtome (Rankin, Wetzlar, Germany) for immunohistochemical staining.

### Immunohistochemistry (IHC)

The slides were placed in the BOND-III instrument manufactured by Leica Biosystems. Default IHC protocol was chosen, and 20 min with epitope retrieval was set as the heat-induced epitope retrieval (HIER) parameter. The Bond™ Polymer Refine Detection (DS9800, Leica Biosystems, Germany) was used for the detection of the primary antibody (anti-ASNS antibody, sc-365809, Santa Cruz, USA). The slides were scanned using Aperio AT2 (Leica Biosystems, Germany) and the digital pictures were viewed using digital slide viewing software in Aperio AT2. The staining intensity was stratified on a scale of 0-3+ (0 = no staining, 1+ = weak, 2+ = moderate and 3+ = strong). An H-score was obtained by multiplying the staining intensity with a constant to adjust the mean to the strongest staining [H-score = 1 × (percent of 1+ cell) + 2 × (percent of 2+ cell) + 3 × (percent of 3+ cell)] to give a score ranging from 0-300. We chose the median of the H-score as the cut-off value for separating patients into two groups: high ASNS expression or low ASNS expression.

### Cell Culture and Small Interfering RNA Transfection

The human chordoma cell line UM-Chor1 was purchased from the American Type Culture Collection (ATCC), another human chordoma cell line MUG-Chor1 was donated by the Chordoma Foundation. Cells were cultured in Iscove’s Modified Dulbecco’s Medium (IMDM, 30-2005, ATCC, USA) supplemented with 18% RPMI-1640 Medium (30-2001, ATCC, USA) and 10% fetal bovine serum (10099-141, Gibco, USA) in a humidified incubator at 37°C in 5% CO_2_. The culture medium was replaced every other day. ASNS small interfering RNA (siRNA) (GGGTAGAGATACATATGGA) and unspecific scrambled siRNA were synthesized by RiboBio Medical Biotechnology (Guangzhou, China). Both UM-Chor1 and MUG-Chor1 cells were adherent cells, the cells were detached from the bottom of the culture flask using trypsin digestion, and this reaction was terminated by adding culture medium, then the cell suspension was centrifuged for 5 minutes at room temperature to collect the cells, finally, the cell pellets were resuspended using culture medium. After counting, 1 × 10^5^ of UM-Chor1 cells and 3 × 10^5^ of MUG-Chor1 cells were seeded in 6-well plates. The cells were transfected with ASNS-siRNA or scrambled siRNA using Lipofectamine 3000 reagent (Invitrogen, USA) and 50 μM of siRNA per well according to the manufacturer’s instructions. After transfecting siRNA, interference efficiency was validated by Quantitative Real-time PCR.

### Cell Growth and Colony Formation Assay

A quantity of transfected cells was seeded into 96-well plates with 2.5 × 10^3^ of UM-Chor1 cells or 6 × 10^3^ of MUG-Chor1 cells per well. Ten microliters of CCK-8 (CK04, Dojindo, Japan) was then added to the plates and incubated for 24, 48, 72 and 96 hours. Upon addition of CCK-8 solution, the plates were incubated at 37°C for 2 hours, and the absorbance was detected at 450 nm using a multimode microplate reader (Tecan, Männedorf, Switzerland). 2 × 10^3^ of transfected UM-Chor1 or MUG-Chor1 cells were inoculated into 6-well plates and incubated at 37°C, 5% CO_2_ for 14 days. Afterwards, the cell colonies in 6-well plates were fixed with 4% paraformaldehyde and stained using crystal violet solution.

### Migration and Invasion Assay

3 × 10^4^ UM-Chor1 cells or 1 × 10^5^ MUG-Chor1 cells transfected with ASNS-siRNA or scrambled siRNA were seeded into the upper chamber of the transwell chambers with 100 μl serum-free medium (CLS3464, Corning, USA), while the following wells were filled with 600 μl complete culture medium containing 10% FBS as a function of chemoattractant which can induce the cells in the upper chamber to the lower one. After incubation for 48 hours, non-invaded cells were rubbed away using the cotton swab carefully, while cells that invaded the lower chamber were fixed with 4% paraformaldehyde and stained using crystal violet solution. We observed the cells that invaded the lower chamber in at least five separate fields of vision using a microscope. The invasion assay was similar to the migration assay but the upper chamber was coated with Matrigel (Corning, USA).

### Protein Extraction and Western Blot Assay

Chordoma tissue samples or cells were lysed using RIPA lysis buffer (C1050, Applygen, China) with a protease inhibitor cocktail (P1265, Applygen, China) and a phosphatase inhibitor cocktail (P1260, Applygen, China). The total protein concentration was determined using a BCA Protein Assay Kit (SK258437, ThermoFisher, USA). Equal amounts of total proteins were separated by SDS-PAGE (10% gels) for ASNS detection. GAPDH was used as the protein loading control. After SDS-PAGE, the proteins on the gels were transferred to BioTrace nitrocellulose membranes (66485, Pall, USA), blocked with 5% skim milk in Tris-buffered saline (TBS, pH 7.4; 20 mM Tris-HCl, 150 mM NaCl), and then incubated with anti-ASNS antibody (1:600, sc-365809, Santa Cruz, USA) overnight at 4°C. The following day, the membranes were incubated with IRDye-labeled goat anti-mouse IgG at room temperature for 1 hour. Finally, the protein bands were scanned using a Li-COR Odyssey system (Li-COR Biosciences, USA). At least three independent experiments were performed and a representative result is shown.

### Statistical Analysis

Statistical analyses were performed using SPSS v24.0 software (IBM Corporation, USA). Variables were analyzed by the chi-square test, Fisher’s exact test, unpaired Student’s *t* test, or Mann-Whitney *U* test for comparison between two groups. Kaplan-Meier curves and the log-rank test were applied for univariable survival analysis. Statistically significant variables were further analyzed by multivariable Cox regression analysis. A nomogram was constructed based on the results of multivariate Cox regression analysis in the training cohort. The nomogram and calibration plots were calculated with the rms package of R software (version 4.2.0). For all statistical analyses, a *p* value less than 0.05 was considered statistically significant.

## Results

### Screening and Identifying the Differentially Expressed Proteins in the Rapid-Recurrence Group and Slow-Recurrence Group of Skull Base Chordoma

**Figure 1:**
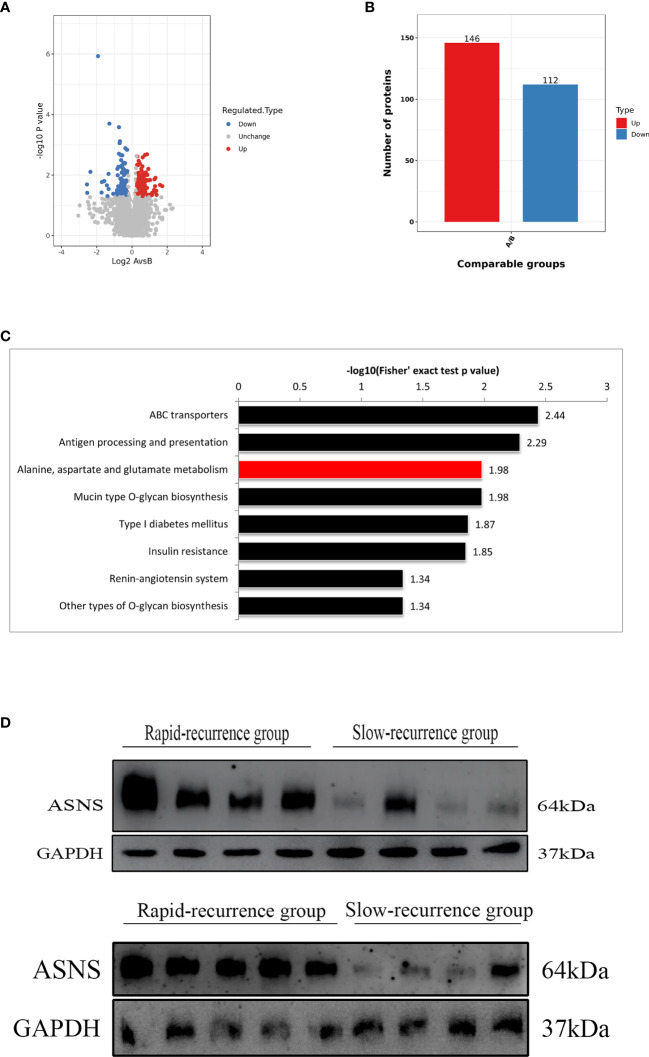
The screening process for proteins upregulated in the rapid-recurrence group of chordoma. **(A)** Volcano plots. The negative log of *p* value (base 10) was plotted on the Y-axis, and the log of fold change (base 2) was plotted on the X-axis. Fold change > 1.25 or fold change < 0.8, *p* < 0.05. A: rapid-recurrence group; B: slow-recurrence group. **(B)** There were 146 proteins upregulated and 112 proteins downregulated in the rapid-recurrence group of chordoma. A: rapid-recurrence group; B: slow-recurrence group. **(C)** KEGG pathway analysis showed that alanine, aspartate and glutamate metabolism was mainly involved in the rapid-recurrence group of chordoma. **(D)** The expression level of ASNS protein in all 17 chordoma tissue samples was quantified by Western blot assay.

To identify proteins that were differentially expressed in the rapid-recurrence group and slow-recurrence group of skull base chordoma, a TMT mass-spectrometric technique was used. 3667 and 3737 proteins were quantified by each technical replicate, respectively, 4286 proteins were quantified from the two technical replicates and subsequently filtered with manually selected filter exclusion parameters. Finally, 258 proteins were screened out, including 146 proteins upregulated and 112 proteins downregulated in the rapid-recurrence group (**Figures 1A, B**). The lists of differentially expressed proteins are shown in **Supplementary Table 5**. Afterwards, 146 upregulated proteins were selected for bioinformatics analysis. Kyoto Encyclopedia of Genes and Genomes (KEGG) pathway analysis revealed that alanine, aspartate and glutamate metabolism was mainly involved in the rapid-recurrence group (**Figure 1C** and **Supplementary Table 2**). Gene ontology (GO) classification also showed that a significant role of glutamine metabolism (glutamine family amino acid metabolic process, glutamine metabolic process) was involved in the rapid-recurrence group (**Supplementary Table 3**). After literature review, ASNS was chosen for validation using Western blot assay. Western blot analysis indicated that the expression of ASNS was significantly upregulated in the rapid-recurrence group (**Figure 1D**), which was consistent with the result of TMT mass spectrometric detection. Thus, we selected ASNS as a tumor marker for IHC to further evaluate its prognosis value in skull base chordoma.

### Relationship Between ASNS Expression and Tumor Recurrence

**Figure 2:**
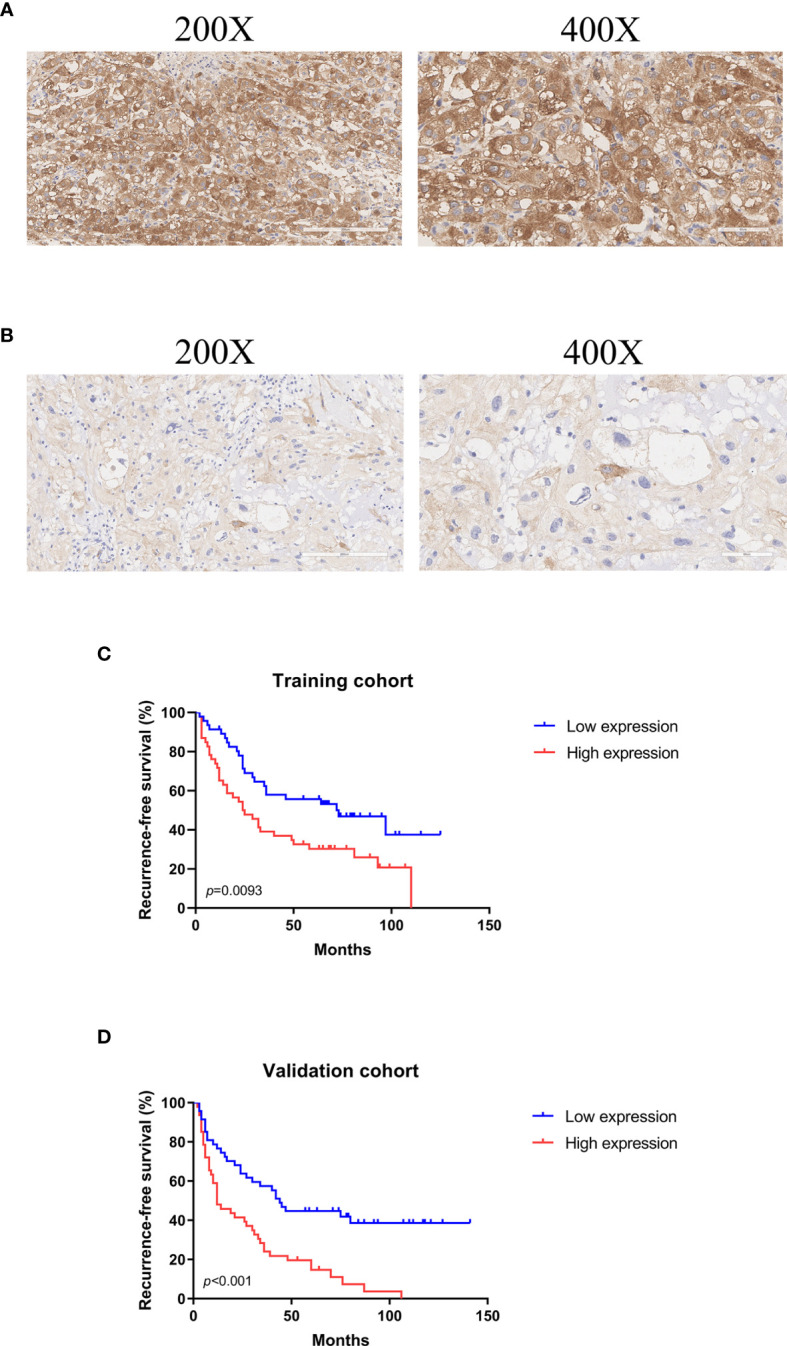
Representative images of ASNS immunohistochemical stain in skull base chordoma and analysis of recurrence-free survival (RFS) using Kaplan-Meier survival curves. **(A)** High expression of ASNS. Magnification: left ×200, right ×400. **(B)** Low expression of ASNS. Magnification: left ×200, right ×400. **(C)** High expression of ASNS was correlated with shorter RFS in the training cohort. **(D)** High expression of ASNS was correlated with shorter RFS in the validation cohort.

ASNS protein was expressed in the cytoplasm and the H-scores of ASNS ranged from 106.27 to 239.58 (median 165.84) (**Figures 2A, B**). For survival analysis, recurrence-free survival (RFS) was defined as the period from the first surgical resection to the date of recurrence or last follow-up. In the training cohort, the median RFS was 32.5 months, and the median RFS in the low ASNS expression group was 59 months, which was 2.4 times as long as that in the high ASNS expression group (24.5 months). Obviously, patients with high expression of ASNS had shorter RFS (median RFS: 24.5 months, 95% CI: 15-49.5 months) compared with patients with low expression of ASNS (median RFS: 59 months, 95% CI: 29.5-68 months), with a *p* = 0.0093 (**Figure 2C**). To further verify the relationship between ASNS expression and RFS, we performed univariate and multivariate Cox regression analysis for RFS in this cohort, and the results revealed that ASNS was an independent prognostic factor for RFS (*p* = 0.042) (**Table 1**). In the validation cohort, the median RFS was 18 months, and the median RFS in the low ASNS expression group was 43 months, which was 3.6 times as long as that in the high ASNS expression group (12 months). Distinctly, patients with high expression of ASNS had shorter RFS (median RFS: 12 months, 95% CI: 9-30 months) compared with patients with low expression of ASNS (median RFS: 43 months, 95% CI: 24-73 months), with a *p* value less than 0.001 (**Figure 2D**). To further verify the relationship between ASNS expression and RFS, we performed univariate and multivariate Cox regression analysis for RFS in this cohort, and the results also revealed that ASNS was an independent prognostic factor for RFS (*p* < 0.001) (**Supplementary Table 4**).

**Table 1:** Univariate and multivariate Cox regression analysis for RFS in the training cohort.

Variables	Univariate analysis			Multivariate analysis		
	HR	95% CI	*P* value	HR	95% CI	*P* value
Age (>55 versus ≤55years)	1.398	0.706-2.770	0.337			
Gender (female versus male)	1.101	0.656-1.848	0.717			
Tumor volume (>20 cm^3^ versus ≤20 cm^3^)	1.949	1.151-3.300	0.013*	1.312	0.749-2.298	0.343
Tumor texture (tough/moderate versus soft)	1.588	0.871-2.896	0.131			
Blood supply (poor/moderate versus abundant)	0.475	0.269-0.836	0.010*	0.495	0.277-0.885	0.018*
Pathology (classical versus chondroid)	1.120	0.641-1.957	0.691			
Extent of resection (non-total versus total resection)	3.740	1.687-8.289	0.001*	3.290	1.410-7.676	0.006*
ASNS (high versus low)	1.960	1.164-3.301	0.011*	1.730	1.020-2.934	0.042*

*indicate p < 0.05.

RFS, recurrence-free survival; HR, hazard ratio; CI, confidence interval.

### Predicting Recurrence by a Nomogram

**Figure 3:**
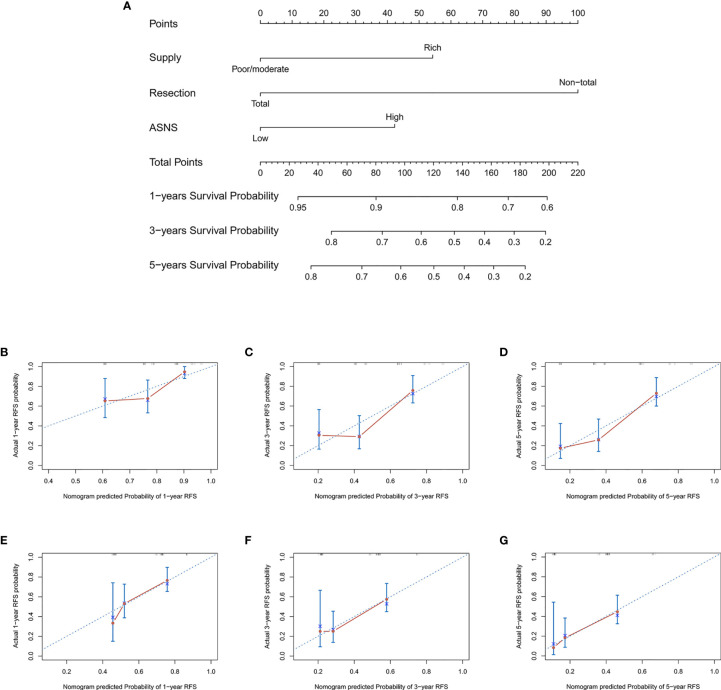
The nomogram for patients with skull base chordoma after surgical resection and the Calibration plots for predicting patient recurrence from the training cohort and validation cohort. **(A)** A nomogram for predicting the probability of recurrence. The calibration curve for predicting recurrence from the training cohort at **(B)** 1 year, **(C)** 3 years, **(D)** 5 years. The calibration curve for predicting recurrence from the validation cohort at **(E)** 1 year, **(F)** 3 years, **(G)** 5 years. Nomogram predicted probability of time-dependent recurrence was plotted on the X-axis; actual recurrence at 1, 3, 5 years was plotted on the Y-axis.

To provide clinicians with a mensurable approach to predict recurrence of skull base chordoma, a nomogram (C-index: 0.720, 95% CI: 0.644-0.797) was constructed using the training cohort data (**Figure 3A**). The calibration plots demonstrated that the nomogram predicted recurrence efficaciously compared with an ideal model in the training cohort (**Figures 3B–D**) and validation cohort (**Figures 3E–G**) at 1-, 3-, and 5-year recurrence.

### Knockdown of ASNS Inhibited Cell Growth, Colony Formation, Migration, and Invasion of Chordoma Cells

**Figure 4:**
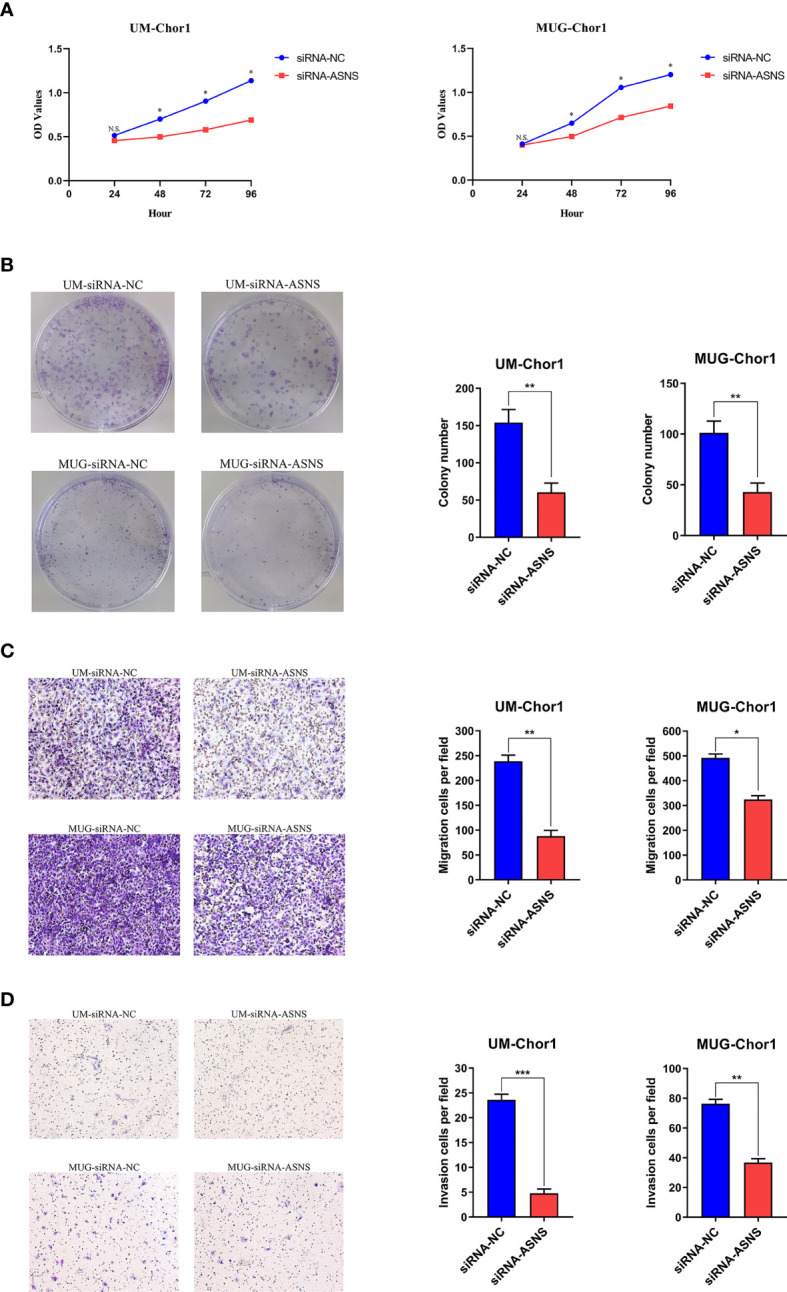
Knockdown of ASNS inhibited cell growth, colony formation, migration, and invasion of chordoma cells *in vitro*. Knockdown of ASNS led to a significant inhibition of **(A)** cell growth, **(B)** colony formation ability, **(C)** migration, **(D)** invasion of UM-Chor1 and MUG-Chor1 cells. Magnification: ×100. Bars represent the mean of the respective individual ratios ± SEM. *p < 0.05; **p < 0.01; ***p < 0.001; ns, not significant.

Based on the clinical data, it seemed that ASNS could affect the proliferation and aggressiveness of chordoma cells. ASNS expression was knocked down in UM-Chor1 and MUG-Chor1 cells by siRNA, the interference efficiency was confirmed at mRNA level by Quantitative Real-time PCR (**Supplementary Figure 1A**) as well as at protein level by Western blot (**Supplementary Figure 1B**). As expected, knockdown of ASNS significantly decreased the growth of UM-Chor1 and MUG-Chor1 cells, as revealed by cell proliferation assay (**Figure 4A**) and colony formation assay (**Figure 4B**). Furthermore, knockdown of ASNS significantly inhibited migration and invasion of UM-Chor1 and MUG-Chor1 cells (**Figures 4C, D**). These findings may explain the above-mentioned TMA results (high ASNS expression in the rapid-recurrence group).

## Discussion

Chordoma is an infrequent, slow-growing, and low-grade malignancy that arises from notochordal remnants, and the annual incidence of chordoma is about 1/1,000,000 (25). Total surgical resection together with adjuvant radiation therapy has been reported to lengthen patients’ survival (26). Using targeted therapies such as imatinib or cetuximab to treat chordoma has been more frequent for the past few years, though these have not involved large amounts of patients with chordoma. The patients have quite poor prognoses for local recurrence and distant metastasis despite comprehensive therapy (3). Consequently, it is in urgent need of exploring the specific molecular mechanisms underlying tumorigenesis of chordoma, searching for novel prognostic factors, and optimizing the existing therapeutic strategies to improve the survival of patients with chordoma.

In this study, chordoma biomarkers were screened using TMT mass-spectrometric technique and bioinformatics analysis. After screening for upregulated proteins in the rapid-recurrence group, a novel prognostic factor was discovered, we found that high expression of ASNS in skull base chordoma was associated with shorter RFS and the results of multivariable Cox regression analysis showed that ASNS was an independent prognostic factor of skull base chordoma for predicting recurrence. *In vitro* investigations have revealed the cancerous function of ASNS in several other tumor types. For example, the ASNS gene has been found to be mutated in human colonic epithelial cells and suggested to be implicated in the initiation of colorectal cancer (27). Additionally, overexpression of ASNS has been reported to be correlated with enhanced aggressiveness in glioma (22). Downregulation of ASNS induces cell cycle arrest in breast cancer cells and inhibits the proliferation (18). Furthermore, a previous study reports that knockdown of ASNS by lentivirus-mediated RNA interference inhibits cell growth in epidermoid carcinoma cells and melanoma cells (17). These results indicate that ASNS plays an important role in oncogenesis, and the results of cytological experiments in our study demonstrate the oncogenic function of ASNS in skull base chordoma.

There are some potential mechanisms by which ASNS causes tumorigenesis. Due to the much higher metabolic requirements in tumor cells and as they are usually grown in the nutrient-deficient environment, transcription and translation of ASNS are activated through some different mechanisms to protect cell survival. For instance, it has been found that mutant p53 binds to the promoter region of the ASNS gene and transactivates its transcription (28). Also, it has been reported that ASNS is upregulated after glucose deprivation and protects pancreatic cancer cells from apoptosis (29). Thus, inhibition of ASNS expression and consequent consumption of asparagine may reduce the proliferative capability of tumor cells. In addition, ASNS has been shown to change the expression of proteins participating in cell cycle progression (30). Cyclin-dependent kinases (CDKs) and cyclins are two kinds of pivotal cell cycle regulatory factors. Cyclin D1, CDK6, and CDK4 are significantly downregulated in human melanoma cells after ASNS depletion while p21, a CDK inhibitor, is obviously upregulated in response to ASNS knockdown (17). Therefore, downregulation of ASNS may impact the cell cycle, resulting in attenuated cell proliferation. Our proteomic data also support this point of view. In our data, we find that CDK18, a cyclin-dependent kinase which plays a role in signal transduction cascades, is upregulated in the rapid-recurrence group, and CDKN1B, a CDK inhibitor, is downregulated in the rapid-recurrence group (**Supplementary Table 5**). However, these detailed mechanisms remain to be further investigated.

Until now, there has been no study of ASNS expression in chordoma. In the present study, we investigate ASNS expression in 187 patients with skull base chordoma by immunohisto-chemistry, and our results demonstrate for the first time that higher ASNS expression in skull base chordoma might indicate a higher recurrence rate. However, further research is necessary on the transcriptional regulation mechanisms of ASNS, also its effect on glutamine metabolism, and its correlation with tumor recurrence and metastasis need to be figured out.

## Conclusion

Our study reveals that high ASNS expression in skull base chordoma is correlated with shorter recurrence-free survival, which supports that ASNS is a novel prognostic factor for predicting the recurrence of patients with skull base chordoma.

## Data Availability Statement

The original contributions presented in the study are included in the article/**Supplementary Material**. Further inquiries can be directed to the corresponding author.

## Ethics Statement

The studies involving human participants were reviewed and approved by the ethics review board of Beijing Tiantan Hospital. Written informed consent to participate in this study was provided by the participants’ legal guardian/next of kin.

## Author Contributions

YS, ML, and CL designed the study. YX, JB, SG, and YZ collected the data. YS and ML analyzed the data and performed the experiments. YS and ML wrote the manuscript. All authors contributed to the article and approved the submitted version.

## Funding

The study was supported by the Research Special Fund for Public Welfare Industry of Health (201402008), the National High Technology Research and Development Program of China (863 Program) and the National Natural Science Foundation of China (30971005).

## Conflict of Interest

The authors declare that the research was conducted in the absence of any commercial or financial relationships that could be construed as a potential conflict of interest.

## Publisher’s Note

All claims expressed in this article are solely those of the authors and do not necessarily represent those of their affiliated organizations, or those of the publisher, the editors and the reviewers. Any product that may be evaluated in this article, or claim that may be made by its manufacturer, is not guaranteed or endorsed by the publisher.
