# Supplementary material for: Proteomics Analysis Identified ASNS as a Novel Biomarker for Predicting Recurrence of Skull Base Chordoma
Source: Front Oncol. 2021 Sep 1;11:698497. doi: 10.3389/fonc.2021.698497 (PMC8440958; doi:10.3389/fonc.2021.698497)

**Supplementary Table 1.** Clinicopathological characteristics of 9 samples of chordoma with short RFS and 8 samples of chordoma with long RFS used in the screening process.

| Factor | Rapid-recurrence group (n=9) | Slow-recurrence group (n=8) | *P* value |
| --- | --- | --- | --- |
| Age (years) |  |  | 0.723 |
| median | 33 | 37.5 |  |
| range | 21-58 | 16-65 |  |
| Sex |  |  | 0.620 |
| male | 5 | 6 |  |
| female | 4 | 2 |  |
| Tumor |  |  | >0.999 |
| primary | 9 | 8 |  |
| recurrent | 0 | 0 |  |
| Bone invasion |  |  | >0.999 |
| limited | 3 | 3 |  |
| extensive | 6 | 5 |  |
| Extent of resection |  |  | 0.725 |
| total | 2 | 2 |  |
| subtotal | 5 | 3 |  |
| partial | 2 | 3 |  |
| Recurrence |  |  | <0.001 |
| yes | 9 | 1 |  |
| no | 0 | 7 |  |
| RFS (months) |  |  | <0.001 |
| median | 3 | 39 |  |
| range | 2-6 | 36-63 |  |

Abbreviations: RFS, recurrence-free survival

**Supplementary Table 2.** Screened candidate by KEGG pathway analysis.


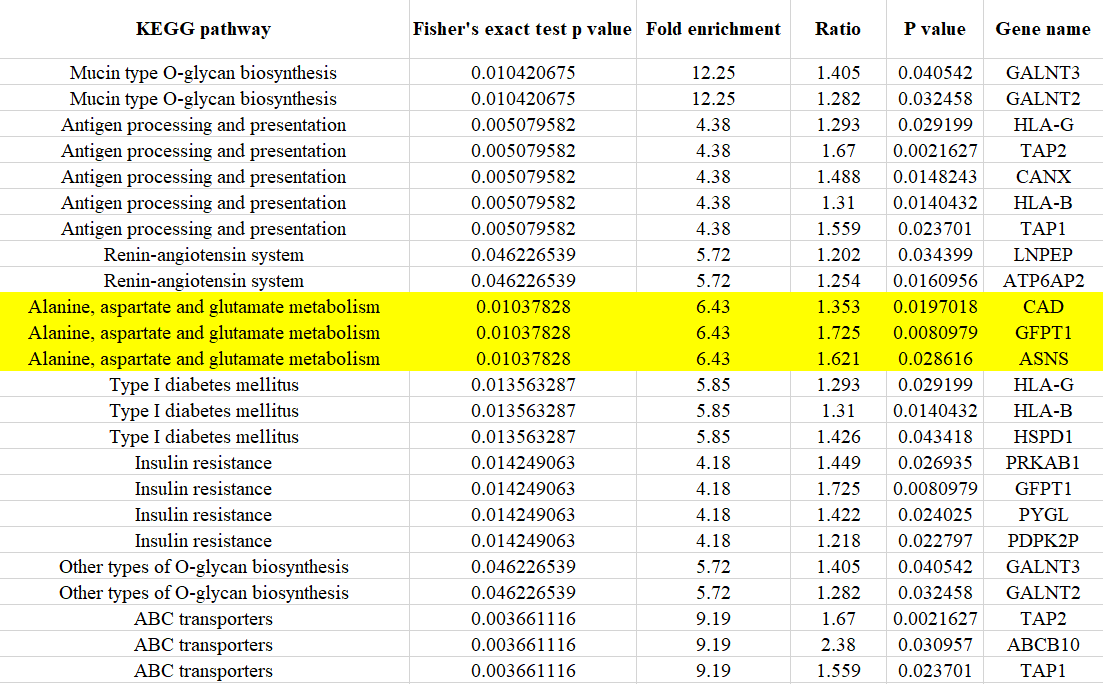


Below was not shown.

**Supplementary Table 3.** Screened candidate by GO classification analysis.


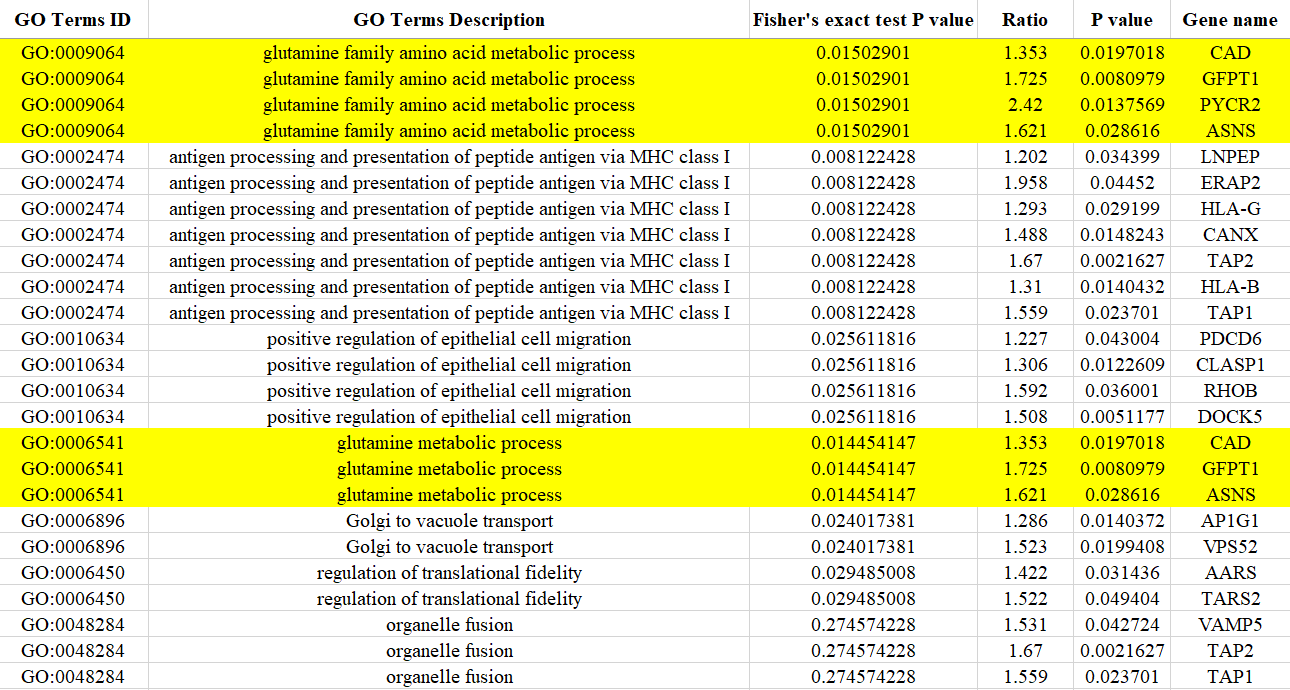


Below was not shown.

**Supplementary Table 4.** Univariate and multivariate Cox regression analysis for RFS in the validation cohort

| Variables | Univariate analysis | | | Multivariate analysis | | |
| --- | --- | --- | --- | --- | --- | --- |
|  | HR | 95% CI | *P* value | HR | 95% CI | *P* value |
| Age (>55 versus ≤55years) | 1.706 | 0.984-2.958 | 0.057 |  |  |  |
| Gender (female versus male) | 1.066 | 0.667-1.704 | 0.790 |  |  |  |
| Tumor volume  (>20 cm^3^ versus ≤20 cm^3^) | 1.464 | 0.906-2.367 | 0.119 |  |  |  |
| Tumor texture  (tough/moderate versus soft) | 1.812 | 1.052-3.121 | 0.032* | 1.997 | 1.147-3.478 | 0.015* |
| Blood supply  (poor/moderate versus abundant) | 0.903 | 0.560-1.456 | 0.675 |  |  |  |
| Pathology  (classical versus chondroid) | 2.283 | 1.325-3.922 | 0.003* | 2.364 | 1.340-4.184 | 0.003* |
| Extent of resection  (non-total versus total resection) | 2.300 | 1.168-4.529 | 0.016* | 1.597 | 0.788-3.236 | 0.194 |
| ASNS (high versus low) | 2.503 | 1.532-4.091 | <0.001* | 2.600 | 1.586-4.262 | <0.001* |

* indicate *p* < 0.05

Abbreviations: RFS, recurrence-free survival; HR, hazard ratio; CI, confidence interval

**Supplementary Figure 1.** Interference efficiency confirmation by (A) real-time PCR and (B) Western blot. Bars represent the mean of the respective individual ratios ± SEM. ***, *p*＜0.001.


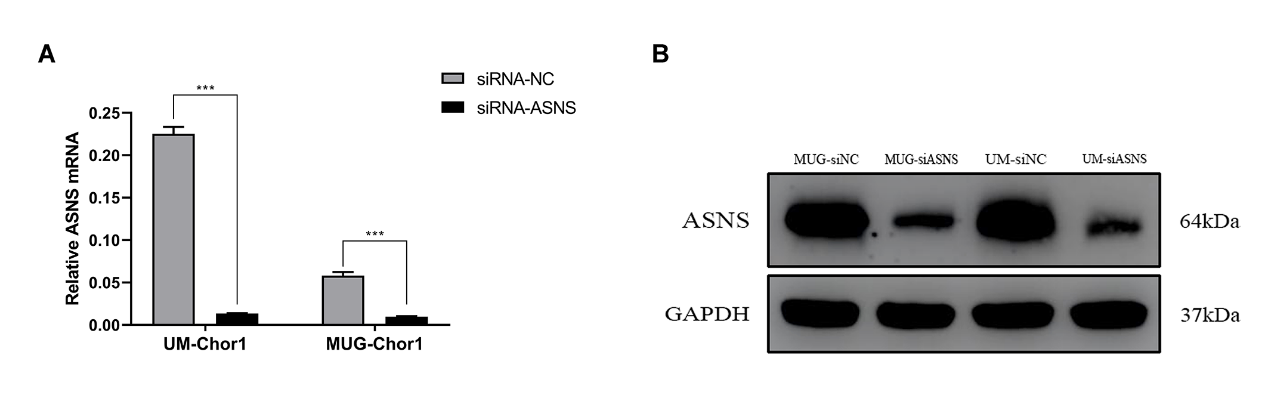

Supplement: Supplementary file 1 [file DataSheet_1.docx]
